# Breed-specific divergence in boar sperm regulatory profiles involves piRNA and mitochondrial small RNAs

**DOI:** 10.1007/s00018-026-06244-8

**Published:** 2026-06-04

**Authors:** Anna Asratian, Signe Isacson, Unn Kugelberg, Colum P Walsh, Anita Öst

**Affiliations:** 1 Department of Biomedical and Clinical Sciences, Linköping University, Linköping, Sweden; 2 Science for Life Laboratory, Linköping, Sweden

**Keywords:** Small non-coding RNA, mitosRNA, Intergenerational responses, miRNA, Livestock breeding

## Abstract

Small non-coding RNAs (sRNAs) act as post-transcriptional regulators and protectors of genomic integrity. Environmental stress and genomic variation can both alter the sRNA composition in sperm, leading to altered offspring phenotypes. Commercial pig breeds, which have undergone years of selective breeding, vary in purpose: some are optimized for meat production, others for high reproductive capacity – traits that are often antagonistic. This study aims to characterize and compare the sperm sRNA composition in four commercial pig breeds, two used for meat production (sire lines) and two for reproductive purposes (dam lines). We find that the two breeds propagated for reproduction traits (Landrace, Yorkshire) show high variability in PIWI-interacting RNA (piRNA) which target transposable elements. In contrast, the two meat production breeds (Duroc, Hampshire) were enriched in mitochondrial ribosomal RNA-derived sRNA (mt rsRNA). These trends may reflect an emphasis on genome protection among reproductive breeds, and on mitochondrial function and metabolic output in meat production breeds. Other notable differences included amongst the miRNAs, which were associated with numerous developmental and metabolic processes. Our study investigates the unique sRNA profiles in boar sperm associated with different breeding objectives, highlighting possible epigenetic mechanisms that could be leveraged in selective breeding programs to optimize fertility, growth and offspring health.

**Supplementary Information:**

The online version contains supplementary material available at 10.1007/s00018-026-06244-8.

## Introduction

Epigenetic factors allow dynamic adaptation to the current environment by regulating gene expression without altering the genetic code. Small non-coding RNA (sRNA) is one class of epigenetic factors acting in germ cells and embryogenesis, and its functions stretch from protection of genomic integrity to inhibiting gene expression [1]. MicroRNAs (miRNAs) were the first sRNA to be identified [2] and are involved in post-transcriptional regulation through binding to Argonaute (Ago) family proteins, that together form the RNA-induced silencing complex (RISC) [3, 4]. Other sRNAs such as transfer RNA-derived small RNA (tsRNAs), ribosomal-derived small RNA (rsRNAs) and PIWI-interacting RNA (piRNAs) can also regulate gene expression through binding to Ago proteins [5–12]. The primary role of piRNAs is to act like a genomic immune system, by binding a subset of Ago proteins to protect against transposable elements (TEs) [13, 14], but they may also regulate some protein-coding genes [14, 15]. The most prevalent TEs in the mammal genome are long interspersed nuclear elements (LINEs), short interspersed nuclear elements (SINEs) and long terminal repeats (LTRs). TEs, also called “jumping genes” due to their ability to move in the genome, are drivers of evolution and diversity. However, they must be tightly regulated to prevent genomic instability and disease. The tsRNAs and rsRNAs, on the other hand, are generated most often as a response to environmental stress [16–19] but can also be influenced by factors such as biological sex [20]. The size and function of these sRNAs vary, and their roles include communication at various levels – from intracellular (e.g. nuclear-mitochondrial interactions [21, 22]) to intercellular [23], and even between hosts [24]. Additionally, a number of other regulatory functions have also been ascribed to these sRNA biotypes [18, 25].

Sperm sRNAs may originate from transcription within the germline genome or be acquired post-testicular via extracellular vesicles such as epididymosomes during epididymal maturation [26–28]. The epididymosomes are mainly carriers of nuclear sRNAs such as tsRNA, rsRNA and miRNA, while the mitochondrial (mt) sRNA are transcribed directly in the spermatozoa [27]. Epididymosomal sRNA content is dynamic and modulated by paternal environmental conditions [26, 29, 30]. Injecting sperm-derived sRNAs – altered in response to environmental stress – into naïve zygotes can reproduce phenotypes similar to those observed in the offspring of fathers that directly experienced the exposure [29, 31, 32]. With these recent discoveries, paternal epigenetic factors in sperm are gaining prominence in fields of intergenerational inheritance and their effects on offspring health [26, 27, 31, 33–35].

The pig has been widely used as a biomedical model for translational studies, including as a model for human reproduction [36], which makes it excellent for investigating intergenerational effects. Pigs have historically been used as essential livestock – they were first domesticated around 9,000 years ago [37]. Over time, humans have selectively bred pigs for desirable traits – such as fertility, and growth rate – laying the foundation for the genetic improvement strategies used in modern pig production. Today, the farming industry maintains nucleus herds to preserve purebred lines, alongside separate multiplier and commercial herds for routine production. The pig breeds in the nucleus herds are often differentiated by purpose – that is, for meat production (the so-called sire lines) or for reproductive capacity (the so-called dam lines) [38]. Dam lines like Landrace and Yorkshire have a reproduction objective i.e. creating sows with optimal mothering abilities, such as large litters, good piglet survival and better nursing traits. Sire lines like Duroc and Hampshire are instead bred to create offspring with meat production traits, such as high meat quality and growth rate. The commercial herds, on the other hand, supply the industry with slaughter pigs, typically produced by crossing a sire line boar and a dam line sow [39].

In pigs, sRNAs have been explored in multiple tissues including the female and male reproductive tracts [40–44]. Studies in muscle tissues have associated miRNAs with processes such as feed efficiency, energy metabolism and muscle formation [45, 46]. As in other species, miRNA are the most extensively studied sRNA and none of the pig studies have examined the whole range of sperm sRNA in detail, nor have they explored breed-specific differences in sperm sRNA content. This study aimed to explore sperm-borne sRNA profiles in four common pig breeds, focusing on difference between meat production and reproductive lines.

## Methods

### Sample collection

Ejaculated sperm (*n* = 24 boars, 6 individuals per breed) from Duroc, Hampshire, Landrace and Yorkshire boars were commercially purchased. Samples were collected by gloved-hand technique and had to reach a motility rate of 70% and not extensively show markers of immature sperm, such as proximal droplets. The quality analysis was performed by Computer Assisted Semen Analysis (CASA) on IVOS II (Hamilton Thorne, MA, USA) before transportation. Sperm samples were diluted (data not shown) based on quality control in CASA and transported in NUTRIXcell + (IMV technologies, L'Aigle, France). Processing of the sperm occurred within 24 h of collection. A drop of semen was visually inspected at arrival for somatic cells in PAULA (Leica Microsystems CMS GmbH, Germany) on a glass slide topped by a cover glass.

At arrival, samples were immediately processed by aliquoting 1 mL sample followed by a 20 min centrifugation at 300* g*. Immediately thereafter the supernatant was removed and 1 mL Qiazol (Qiagen, Venlo, Netherlands) was added together with one 5 mm Stainless steel Bead 0,5 mm bead (Qiagen, Venlo, Netherlands). Samples were homogenized in the TissueLyzer for 2 min in 40 oscillations followed by a 5 min incubation, twice. Thereafter, the sample was stored in the −80 ^o^ C freezer until RNA extraction.

### RNA extraction

Total RNA was extracted with miRNeasy Micro kit (Qiagen, Venlo, Netherlands) according to the manufacturer’s instructions. The homogenized sample previously mixed with Qiazol was thawed in room temperature. Chloroform (ThermoFisher Scientific, MA, USA) addition was followed by 15 s of shaking. After a short (2 min) incubation in room temperature, the samples were centrifuged for 15 min at 12 000 g in 4° C. The aqueous phase was mixed with 100% ethanol. Thereafter, samples were transferred to columns provided by the kit and washed according to the protocol prior to elution with 14µL RNase free water. Samples were quality controlled on Agilent 2100 Bioanalyzer version (Agilent Technologies, Ketch, Germany) on RNA 6000 Pico kit (Agilent Technologies, Ketch, Germany).

### Small non-coding RNA sequencing

The NEBNext Small RNA Library Prep Set for Illumina (New England Biolabs, Ipswich, MA) was used according to the manufacturer’s instructions with a minor alteration of diluting the primers from the kit 1:2. Libraries were further cleaned with AMPure XP Bead-based Reagent (Beckman coulter, CA, USA) and concentration was measured with Quantus fluorometer (Promega, WI, USA). The quality of the samples was examined with a High Sensitivity DNA kit on Bioanalyzer (Agilent Technologies, Ketch, Germany). Next, 5,5 ng of each library was pooled and loaded to a 6% polyacrylamide Novex TBE gel (Invitrogen, MA, USA). Size selection (16—75nt) was manually made, and the libraries were eluted using included buffer. Libraries were precipitated determined with QuantiFluor ONE dsDNAsystem on a Quantus fluorometer (Promega, Madison, WI). Size and concentration were confirmed using a High Sensitivity DNA kit on the Bioanalyzer. Then, the libraries were sequenced on NextSeq 550 with NextSeq 500/550 High Output Kit version 2, 75 cycles (Illumina, San Diego, CA).

### Bioinformatic analysis

All preprocessing was performed with Seqpac version 1.2.0 in R version 4.3.2. Adaptors (AGATCGGAAGAGCACACGTCTGAACTCCA) were trimmed out from the sequence reads in R (version 4.3.2). The sequence reads included in the analysis were restricted to those with an adaptor present, within 16—75nt of length and an Illumina quality score of > 20 in 80% of the nucleotides. Unique sequences were then counted and data was filtered to have a read threshold of 5 read in 10% of the samples before normalization with counts per million (CPM). Additional filtering of 1 CPM in 5% of all samples were performed after normalization.

The references used for mapping each respective breed was Duroc (GCF_000003025.6), Landrace (GCA_001700215.1), Hampshire (GCA_001700165.1) and Large White (GCA_001700135.1), which is the closest available genome to Yorkshire. For the sRNA biotypes, Ensembl ncRNA for Sscrofa11.1 for ncRNA, piRBase ssc.v3.0 for piRNA and GtRNAdb sus-Scr11-mature for tRNA were used. In all mapping, a mismatch of 3 was allowed to account for possible breed disparities in sRNA annotation databases.

For RepeatMasker analysis, RepeatMasker version 4.0.7 was used [47], running following command on all four genomic references (GCF_000003025.6, GCA_001700215.1, GCA_001700165.1, GCA_001700135.1):-species pig –gff -html.

We used the miRNA database miRDB (http://mirdb.org/) [48] to identify miRNA targets to the top 5 upregulated miRNAs in our analysis. The gene symbols equal to or above 90 as a Target Score from hsa-miR-191—5p, hsa-let-7a-2—3p, hsa-miR-9—3p, hsa-miR-9—5p, hsa-let-7i-5p, hsa-let-7f-2—3p was entered into STRING (https://version-12-0.string-db.org/organism/9823) with *Sus scrofa* set as organism for a gene ontology analysis [49].

Seqpac version 1.2.0, ggplot2 version 3.5.0, UpsetR version 1.4.0 and pheatmap 1.0.12 were used to visualize the RNA-seq data. Factoextra version 1.0.7 and FactoMiner version 2.10 were used for performing and visualization of principal component analysis. Circlize version 0.4.16 was used for visualizing miRNA chromosomal origin. All code for the full analysis is available https://github.com/signeisacson/Asratian_Breed.

For overview analysis of genomic occupation of repeats (Fig S2), summary table (Table S1) provided by RepeatMasker was used. No multimapping was allowed in the RepeatMasker analysis.

### RT-qPCR

Four technical replicates from a Duroc (sire line) and a Yorkshire (dam line) sample were used for the RT-qPCR analysis. We performed the analysis with stem-loops as described by Chen et al. [50] and Kramer [51] with the longer reverse primer introduced by Kramer [51]. To normalize the miRNAs an identifiable miRNA (MIR10B) was selected from the sequences that had the most stable CPM across all 24 samples. This was calculated by the Coefficient of variation (CV) for each sequence. All primers were manually designed, and Tm was tested in Primer express (version 3.0, ThermoFisher). All sequences used are available in Table S2. Stem-loops were ordered through Custom Standard DNA Oligos (ThermoFisher Scientific, MA, USA), while the forward/reverse primers and probe were ordered by Sequence Detection Primer (ThermoFisher Scientific, MA, USA). The reverse transcription was run using TaqMan™ MicroRNA Reverse Transcription Kit (ThermoFisher Scientific, MA, USA) with 0,5 µM of each stem-loop. In the quantification step we used TaqMan™ Fast Advanced Master Mix for qPCR (ThermoFisher Scientific, MA, USA) with 0,9 µM of each primer (forward/reverse) and 0,25 µM probe. The cycle threshold (Ct) values of reference miRNA (Stable sequence) were subtracted from the expression of each target miRNAs (mir-191 (upregulated) and mir-28 (downregulated)) for Δ Ct. The average of the sire line samples for each miRNA was subtracted from all Δ Ct, producing ΔΔ Ct values. Unpaired t-tests were performed on these values. Thereafter, fold change was calculated by the formula: 2 ^ (- ΔΔ Ct).

### Statistics

Comparison between breeds and lines were performed and visualized in IBM SPSS Statistics version 29 and GraphPad Prism 10.0.2 (GraphPad Software). One-way ANOVA with Bonferroni post hoc test or otherwise Kruskal–Wallis H test with Dunn’s test for pairwise comparisons was used for breed differences. Differences between lines were analyzed by Unpaired t-test or Mann–Whitney U-test. *P*-values (2-sided) less than 0,05 were considered significant, and in the differential expression analysis adjusted p-values less than 0,1 were considered significant. Error bars depict SEM. Microsoft® Excel® (for Microsoft 365 MSO (Version 2312 Build 16.0.17126.20132) 32-bits) was also used for data processing.

## Results

### Sperm sRNA composition in the major commercial pig breeds

**Fig. 1:**
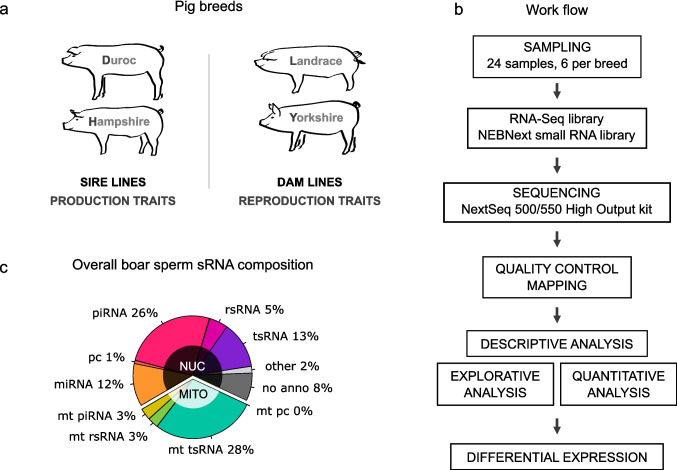
Isolation and initial characterization of boar sperm sRNA. **a** Sperm from four common pig breeds (Duroc, Hampshire, Landrace and Yorkshire) was collected by the gloved hand technique. **b** The sperm samples (*n* = 6 per breed) were assessed for quality; total RNA was extracted and then a size-selection step (16—75nt) was performed before libraries were prepared for analysis on a NextSeq 550. Quality control of output and a sequence-based bioinformatic analysis was performed using Seqpac in R. Following quality control and explorative analysis including quantitation, groups were identified and differential expression examined. **c** The overall breakdown of the sRNA composition in an average sample. miRNA = microRNA; mt pc = mitochondrial protein coding; mt piRNA = mitochondrial PIWI-interacting RNA; mt rsRNA = mitochondrial ribosomal RNA-derived small RNA; mt tsRNA = mitochondrial transfer RNA-derived small RNA; no anno = no annotation; other; pc = protein coding; piRNA = PIWI-interacting RNA; rsRNA = ribosomal RNA-derived small RNA, tsRNA = transfer RNA-derived small RNA

We investigated the sRNA profile in porcine sperm from four commercial breeds (six boars per breed) used in the Swedish pig farming industry (Fig. 1a, Table S3): Duroc (D), Hampshire (H), Landrace (L) and Yorkshire (Y). The first two breeds are defined as sire lines as the purpose is to create offspring for meat production purposes (e.g. high meat quality), while the latter two are dam lines prized for their reproductive qualities (e.g. good mothering ability, large litter size). Figure 1b illustrates the workflow after arrival of samples: RNA extraction was performed with a commercial kit prior to library preparation using a protocol specific for sRNA, with some modification (see methods for details). Libraries were sequenced on a NextSeq550 and post-sequencing analysis done using the Seqpac workflow developed in-house and described elsewhere [52].

Comparison of sperm samples indicated no statistical differences between breeds regarding sperm concentration, with an average of 29 million spermatozoa per mL (Table 1). Visual inspection of the samples revealed no presence of somatic cells, either by microscopy or by Bioanalyzer 2100 (Agilent, CA, USA). RIN values ranged between 2.6—2.8, and the yield of sRNA per spermatozoa averaged at 1.14 fg (range 0.16—2.8 fg), similar to previous findings [53]. No statistical differences in RNA yield were found between the breeds. RNA sequences between 16 and 75nt in length following adaptor removal were matched to sRNA biotypes using specific databases as indicated in methods.

**Table 1:** Background data on sperm samples

Breed	Concentration (million/mL)	RIN value	Concentration Bioanalyzer (pg/µL)	Total sRNA (ng)
Duroc	28.4	2.6	5 135.7	22.4
Hampshire	27.7	2.7	8 055.7	34.0 *
Landrace	31.5	2.8 **	10 183.3	31.8
Yorkshire	28.2	2.6	7 169.0	35.0

All but the last category (Total sRNA) had normal distribution, in those average levels from all individuals are shown. In the case of the non-normal distribution (Total sRNA in Hampshire), median is depicted. No significant differences were found. * non-normal distribution, ** too small sample size

Analysis indicated that overall, about a third of the boar sperm sRNA profile is of mitochondrial (mt) origin, while two-thirds are nuclear (Fig. 1c). Much of the mitochondrial fraction consists of transfer RNA-derived small RNA (tsRNA), accounting for 82% of the mitochondrial fraction and 28% of the overall sample. In the nuclear fraction, the main components were piRNA (26%) and tsRNA (13%). Combined, tsRNA of mitochondrial and nuclear origin together account for 41% of the total sRNA profile, by far the largest single component, with piRNA being the next most prevalent biotype (29% combined). Other significant components are miRNA at 12% and sRNA originating from ribosomal RNA (rsRNA) at 8% in total (5% nuclear and 3% mitochondrial). For this analysis, up to 3 mismatches were allowed, and only 8% of the sRNA could not be unequivocally assigned. Using stricter criteria (no mismatches), 30% of the sRNA were unannotated, but tsRNA and piRNA were still the largest components of the population (Fig. S1a), indicating that a more relaxed match is not substantially skewing the data, while allowing us to identify more of the component RNA.

### Variability in piRNA, mitochondrial sRNA and miRNA distinguishes sample groups

**Fig. 2:**
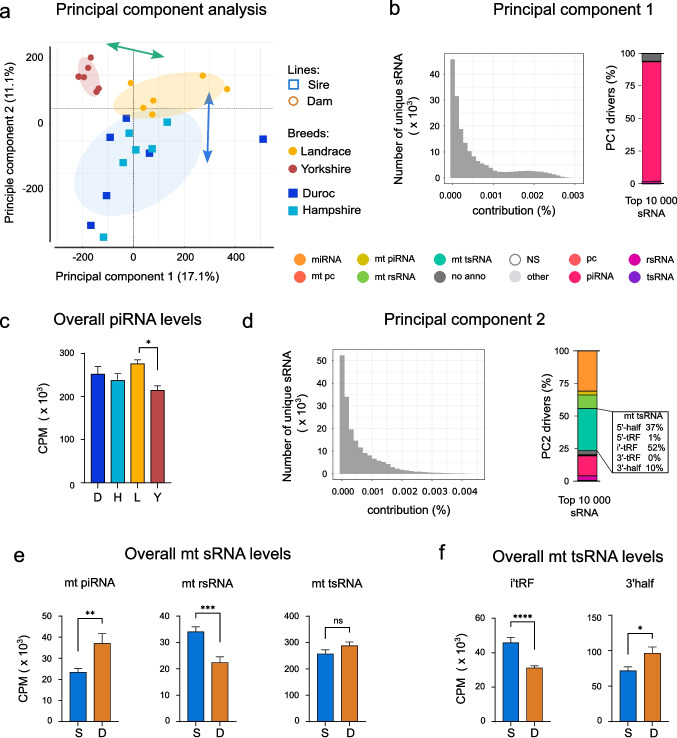
piRNA and mitochondrial sRNA are the main drivers of diversity. **a** A principal component analysis based on sRNA data of all sperm samples. Green arrow points to the separation of dam line breeds, while blue arrow points to differences between breed objectives (sire vs dam lines). Both arrows are added manually. **b** Breakdown of principal component (PC) 1 by a scree plot visualizing the percent contribution of sRNAs and a stacked bar of the top 10 000 sRNAs. **c** One-way ANOVA on overall piRNA levels (mean CPM) between breeds. **d** PC2 illustrated by a scree plot with contribution of each unique sRNA and a stacked bar of the top 10 000 sRNAs. The box depicts a further breakdown of mitochondrial tsRNAs (also known as tRFs). **e** Comparison of overall sRNA levels (mean CPM) in the mitochondria. Unpaired t-test used for mt rsRNA and tsRNA, while mt piRNA was analyzed by Mann–Whitney test. **f** Comparison of overall mitochondrial tsRNA levels (mean CPM) by Mann-Whitney test. Abbreviatios, see Fig. 1. Additional: D = Duroc, H = Hampshire, L = Landrace, Y = Yorkshire; S = Sire lines, D = Dam lines; i’-tRF = internal fragments of tRNA, tRF = tRNA-derived sRNA fragments. *p< 0.05, **p< 0.01, ***p< 0.001, ****p< 0.0001

To investigate the sources of variability arising in the dataset, a principal component analysis (PCA) was carried out using the sRNA data from the individual boar samples (Fig. 2a). We found three clusters, two of which are separated on PC1, and a third that separates on PC2. The top two principal components account for 17.1% (PC1) and 11.1% (PC2) of the variability between them (Fig. 2a). PC1 largely separates the two dam lines Yorkshire (red ellipse) and Landrace (yellow ellipse) from each other, whereas PC2 separates the two sire lines (blue ellipse) from the dam lines with only a slight overlap.

To understand the main drivers involved, we analyzed the contribution of each sRNA to PC1 using a scree plot (Fig. 2b, left, Table S4). We then explored the biotypes present in the top 10 000 drivers (Fig. 2b. right), which were almost exclusively piRNA (pink), with the main remaining contribution from non-annotated sequences. This suggests that the variability between dam lines is driven by the diverse piRNA content of the sperm. To confirm this using a separate approach, we compared piRNA levels by normalized read counts (CPM) – between the breeds, which again revealed significant differences between the two dam lines (Fig. 2c).

We also looked for the main drivers of variance in PC2 using a scree plot (Fig. 2d. left, Table S5). This showed more diversity amongst the top 10 000 drivers than PC1 (compare Fig. 2d to b). There was a large contribution from miRNA (Fig. 2d. right, orange) but in fact the largest single biotype represented here consisted of tsRNA (Fig. 2d. right, mint green), almost exclusively derived from the mitochondrial and not the nuclear tRNA. The next biggest driver was nuclear piRNA (pink). A substantial fraction also consisted of mt rsRNA (green). Overall, mitochondrial-derived sRNA biotypes (shades of green in Fig. 2d) were the major contributors to PC2, suggesting this distinguished sire and dam lines.

Consistent with this, an independent analysis of overall levels of sRNA derived from mitochondria showed significant differences between sire and dam lines for both piRNA and rsRNA, though interestingly not for tsRNA (Fig. 2e, Fig S1b). As this latter result seemed to contradict the PC2 findings (Fig. 2d), we looked more closely at the mitochondrial tsRNAs (also known as tRFs). This indicated that the largest fraction (52%) of these sRNAs match internal fragments (i’-tRF) of tRNA, with 5’ halves (37%) and 3’ halves (10%) next (Fig. 2d. boxed). Comparing the overall mt tsRNA levels in more detail, we found that both i’-tRF and 3’ halves of mitochondrial tsRNA did in fact show significant differences, particularly the former (Fig. 2f). The 5’ halves of tsRNA did not show significant differences in overall count (not shown). This closer analysis therefore also supported a role for these mitochondrially-derived fragments in differentiating breeds used for meat production versus reproduction.

### Dam lines have a diverse set of piRNA

As our analysis of overall differences in sRNA levels and variance indicated distinct separation between the dam and sire lines, we continued by investigating this using a differential expression (DE) analysis comparing the two dam lines to the two sire lines.

**Fig. 3:**
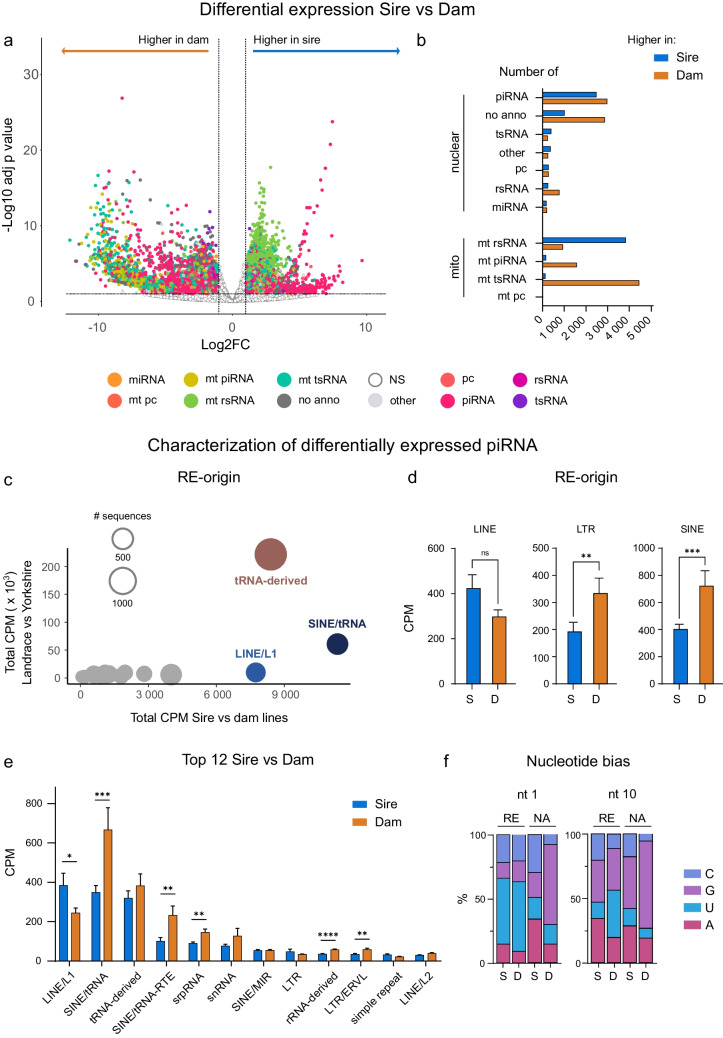
piRNA in dam lines target SINEs and LTRs. **a** Differential expression (DE) analysis by DEseq2 comparing sRNA data in sire vs dam lines. **b** Number of sRNAs from respective biotypes that are differentially expressed comparing sire to dam lines. **c** Characterization of the differentially expressed piRNAs by RepeatMasker. X-axis depicts the results (total CPM) from the sire to dam line comparison, while y-axis depicts the intra-dam comparison (total CPM; Yorkshire vs Landrace). The size of the dot represents the number of sRNAs in that group. **d** Comparison (mean CPM) of DE piRNAs associated with the 3 most common repetitive element (RE) classes in the pig genome (LINE, SINE and LTR) between sire and dam lines by Mann–Whitney U-test. **e** Sire and dam line levels of piRNAs originating from respective RE families. Mann–Whitney U-test was performed within each RE-family. **f** Nucleotide bias at 1st and 10th position on up- (S) or downregulated (D) piRNAs associated with REs (REs) or not associated (NA). Abbreviations, see Fig. 1. Additional: LINE = Long interspersed nuclear element; LTR = long terminal repeat elements; rRNA = ribosomal RNA; SINE = short interspersed nuclear elements; snRNA = small nuclear RNA; srpRNA = Signal recognition particle RNA; tRNA = transfer RNA. D = Duroc; H = Hampshire; L = Landrace; Y = Yorkshire. *p<  0.05, **p< 0.01, ***p< 0.001, ****p< 0.0001

Figure 3a shows log2 fold change (FC) distributed by adjusted p-value (adj p value) of sRNA in sire vs dam lines (Table S6). Here again differences can be seen between the two types of breeding objectives, with piRNA and mitochondrial biotypes of sRNA being most prevalent, reflecting differential expression. Looking at this in terms of numbers of sequences (Fig. 3b), there are almost equal numbers of nuclear piRNA upregulated in either sire or dam lines, indicating that it is not the presence or absence of piRNA, but rather different types of piRNA being present in the two lines (see Fig. S2a, b for comparison between dam line breeds (Table S7)). In contrast, there were far higher numbers of mt rsRNA in sire line breeds than dam line breeds (Fig. 3b, bottom), whereas the opposite was true for mitochondrial tsRNA.

While they have been co-opted for many secondary purposes, the main function of piRNA is protecting the genome against invasion by transposable elements (TE), which are a category of repetitive elements (REs) [13, 14]. On this basis, we therefore explored which REs the piRNA from each breed originate from. Hampshire, Landrace and Yorkshire genomes had no available annotations on the RepeatMasker Genomics Dataset (Institute for Systems Biology). For that, we produced reference files using RepeatMasker for all four breeds and found the genomic breakdown of REs to be relatively similar between the breeds (Fig. S2c). Based on the reference genome Duroc, the total proportion of the pig genome consisting of REs was 43.89%, of which LINE, LTR elements and SINE accounted for 20.52%, 4.59% and 14.71%, respectively.

We then proceeded to map the significant piRNA from the differential expression analysis in Fig. 3a to RE families with RepeatMasker. As can be seen from Fig. 3c, piRNAs which mapped to SINEs showed the greatest overall difference between sire and dam lines (x-axis) along with those mapping to LINEs. While tRNA-derived piRNA (Fig. 3c, brown, Table S8) also displayed large differences between sire and dam lines, the variation between the two dam lines (y-axis) was even greater. This suggests that these piRNAs may play a lesser role in distinguishing reproduction and production breeds. Consistent with these findings, there was a significant difference in levels of SINE-associated piRNAs between dam and sire lines (Fig. 3d, see further detailed breakdown in Fig. 3e), suggesting an important role for this sRNA biotype in reproduction breeds. Likewise, LTR-originating piRNA were also significantly over-represented in dam lines (namely Landrace, Fig. S2d). While we found a trend (*p* = 0.052) of higher levels of LINE-originating piRNA in sire than dam breeds, the difference was not significant (Fig. 3d). Overall, therefore, dam lines exhibited a greater prevalence of piRNAs derived from specific RE families, in particular SINEs and LTRs, suggesting a potential breed-related enrichment.

Clusters of piRNAs involved in the suppression of RE are transcribed and cleaved by members of the mammal phospholipase D (MitoPLD) family, resulting in a characteristic 1U bias [54]. By calculating the 1U bias occurrence in piRNAs associated with REs (RE) or not (NA), we found that there was a clear preference for such a bias in all piRNA which have been mapped to REs in both sire and dam lines (Fig. 3f. right), as expected. We did not find a clear 10A bias in any of the groups, though the RE-associated piRNAs in sire lines show a slight trend towards it (Fig. 3f).

Our analysis so far therefore suggests a difference in piRNA levels between dam and sire lines, with dam lines showing higher amounts and greater variability. Most of the piRNAs are likely to be actively involved in RE suppression, particularly of LINEs, SINEs and LTR-containing elements, which are the major classes which are transposed and cause genomic instability, but further targeted studies are needed to prove this hypothesis.

### Sire lines show enrichment for mitochondrial rsRNA

**Fig. 4:**
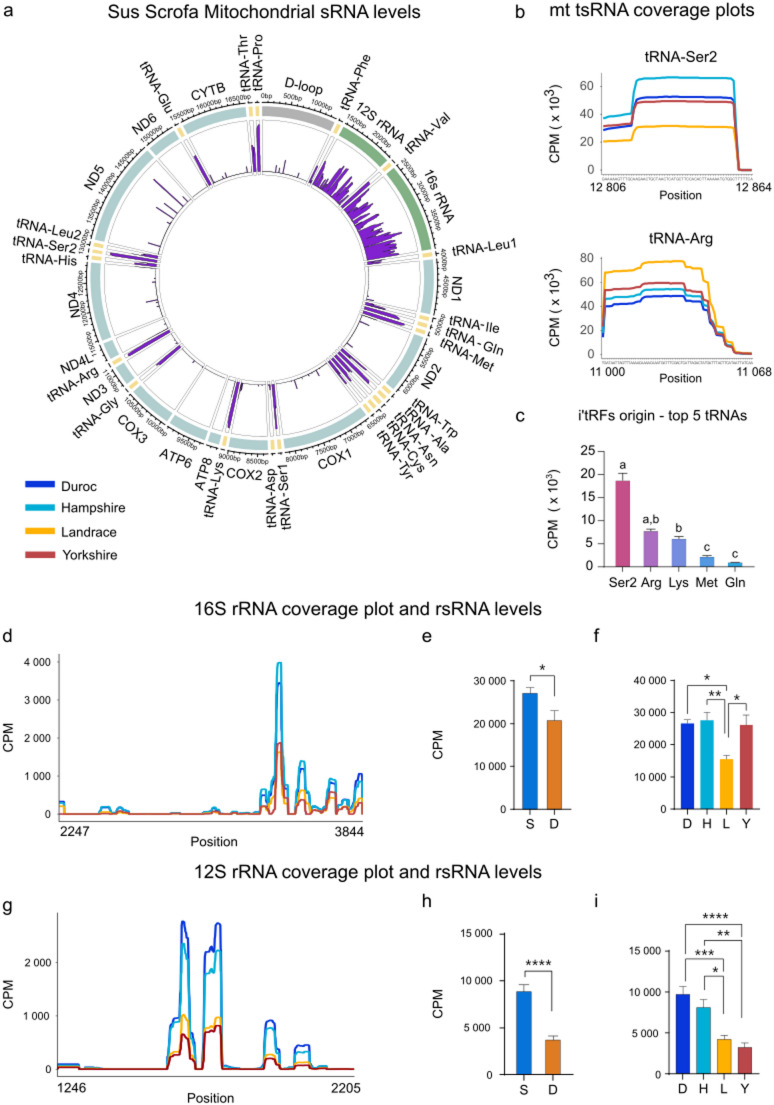
Higher levels of mitochondrial rsRNA in sire lines. **a** Porcine mitochondria with average expression of sRNAs shown in purple. **b** Coverage plots of top 2 tRNAs. **c** The tRNA-origin of the tsRNA from PC2 top 10 000 sRNAs (Fig. 2d). A Kruskal-Wallis test was performed and shared letter (a-c) indicate no significant difference. **d** Coverage plot of 16S rRNA from top 10 000 drivers of PC2. **e** Breed comparison of 16S rsRNA levels (mean CPM) by Unpaired t-test. **f** Comparison of 16S rsRNA levels (mean CPM) between sire and dam lines by One-way ANOVA. **g** Coverage plot of 12S rRNA from top 10 000 drivers of PC2. **h** Breed comparison of 12S rsRNA levels (mean CPM) by Unpaired t-test.** i** Comparison of 12S rsRNA levels (mean CPM) between sire and dam lines by One-way ANOVA. Abbreviations, see Fig. 1. Additional: Arg= Arginine, Gln= Glutamine, Met= Methionine, Lys= Lysine, Ser2= Serine-AGY (position: 12 806-12 864). * p < 0.05, ** p < 0.01, *** p <0.001.

Mitochondrial sRNA accounted for the major differences between sire and dam lines in the overall analysis of variance in the PCA (Fig. 2a, d-f). When examining differential expression between dam and sire lines, both the mitochondrial tsRNA and rsRNA displayed clear differences (Fig. 3a-b). To explore this further, we first plotted the sRNA levels present in all samples to where they map on the mitochondrial circular genome (Fig. 4a, purple coverage plot in inner circle). As shown in Fig. 4a, sperm-borne sRNA primarily originates from rRNA and tRNA sequences, showing that, apart from the high levels of 12S and 16S rsRNA, there were also high levels corresponding to specific tRNA. We then investigated the breed-specific profiles of individual tRNA (Fig. 2d, Fig. S3). While some tsRNA showed higher levels in sire lines, such as tRNA-Ser2 and some lower, such as tRNA-Arg (Fig. 4b), many other tRNA did not show a split along dam/sire lines (see for example tRNA-Lys, Fig. S3). Furthermore, the i'tRF, which were one of the major drivers of the dam-sire split (Fig. 2d, f), predominately arise from Ser2 and Arg (Fig. 4c), suggesting differences in these tRNA between dam and sire lines. There was also a clear difference in 16S rsRNA levels between sire and dam (Fig. 4d), with sire lines having much higher levels. Averaging overall CPM confirmed that sire lines generated levels that were significantly higher than those seen in dam lines (Fig. 4e). This however was not as clear between breeds (Fig. 4f). Likewise, 12S rsRNA levels were also higher in sire lines (Fig. 4g) and these differences were statistically significant compared to dam breeds (Fig. 4h), which could also be seen at the breed level (Fig. 4i).

### Upregulated miRNAs are linked to developmental and metabolic processes

**Fig. 5:**
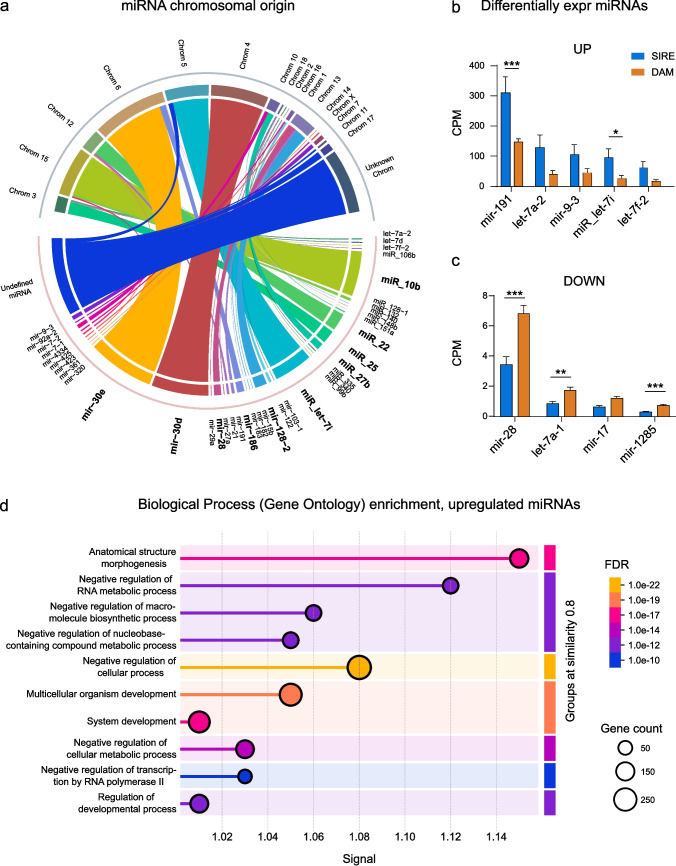
Upregulated miRNAs are involved in developmental and metabolic processes. **a** Chromosomal origin of all miRNAs with an average CPM above 10 of all samples identified as top 10 000 drivers of PC2 (Fig. 2d). The top 10 most prevalent miRNAs are depicted in bold. The thickness of the band represents CPM. **b-c** Levels (mean CPM) of up- and downregulated miRNAs from sire vs dam line DE analysis that are also in the top 10 000 drivers of PC2. An Unpaired t-test or Mann–Whitney U-test was performed within each miRNA. **d** Gene ontology enrichment analysis with STRING. Signal incorporates observed and expected genes with false discovery rate (FDR). *p < 0.05, ** *p* < 0.01, ***p < 0.001

Part of the separation of breeding objectives in our PCA plot (Fig. 2a, d) was due to miRNAs, which have been reported to play key regulatory roles. We therefore investigated which biological processes the specific miRNAs differentiating sire from dam lines are associated with. First, we mapped the miRNAs, which were top drivers of PC2, to their chromosomal location (Fig. 5a, Table S9). These miRNAs included top 10 highest expressed: mir-30e, mir-30d, microRNA-10b, microRNA-let-7i, microRNA-22, microRNA-25, mir-128-2, mir-28, mir-186 and microRNA-27b (all depicted in bold). The differences in origin indicated that the miRNAs contributing to the differences in sire and dam lines did not come from miRNA clusters. We continued by extracting only those miRNAs found in the differential expression analysis (Fig. 3a) from the top 10 000 drivers in PC2 (Fig. 2d). Figure 5b shows the top 5 upregulated miRNA (mir-191, let-7a-2, mir-9-3, miRNA-let-7i, let-7f-2) out of a total of 35 with CPM above 0. In contrast, only four miRNA were identified of the downregulated miRNAs (mir-28, let-7a-1, mir-17 and mir-1285) and these all had low CPM (Fig. 5c). The expression levels of the top up- and down regulated miRNAs in this analysis were measured by RT-qPCR in Duroc and Yorkshire samples, confirming the expression differences between the breeding objectives (Fig. S4).

As the downregulated miRNAs are very lowly expressed, we focused on the top 5 up-regulated miRNA and performed a gene ontology enrichment analysis with STRING (version 12.0, https://string-db.org/) in their predicted targets. This suggested associations (Fig. 5d) in developmental pathways such as anatomical structure morphogenesis (FDR 1 × 10^–17^) and multisystem organism development (FDR 1 × 10^–19^). The upregulated miRNAs were also predicted to be involved in metabolic control, such as negative regulation of RNA metabolic processes (FDR 1 × 10^–12^) and negative regulation of transcription by RNA polymerase II (FDR 1 × 10^–10^), though at lower significance rates than the developmental pathways (Fig. 5d).

## Discussion

Here, for the first time, we compared the sperm-borne sRNA from four pig breeds commonly used for commercial purposes, namely Duroc, Hampshire, Landrace and Yorkshire, two of which are called sire lines and have a meat production focus (Duroc, Hampshire) and two with a focus on reproductive capacity (Landrace, Yorkshire), called dam lines. Our explorative analysis indicates that piRNA and mitochondrial sRNA, along with miRNA, were the three main sources of diversity between breeds. The two dam lines show high diversity in piRNA, while mitochondrial rsRNA showed higher levels in the sire lines. The piRNAs appear to target LTRs and SINEs in dam lines. The miRNA populations differ between sire and dam lines, and we could associate developmental and metabolic processes targeted by the upregulated miRNAs.

Paternal intergenerational inheritance is still an emerging field, with new sperm-borne sRNAs being identified as influencing the phenotype of the next generation. These effects have been studied by microinjecting miRNAs [55–57], and tsRNAs [29, 32] or rsRNAs [58] into naïve zygotes and assessing the resulting offspring phenotypes. Interestingly, the biological sex of the offspring modulates the phenotypic effects of the paternal RNA profile [31, 59], which may have implications for livestock breeding. Although sparsely investigated, sperm sRNA profiles may influence economically important traits in the offspring, such as meat quality from sire line boars and maternal ability from dam line boars. Supporting evidence in cattle associates certain sperm-borne tsRNAs and miRNAs with fertility in female offspring [60].

PIWI-interacting RNAs (piRNAs) have a primary function in host genome defense from transposable elements (TE) and are important for germ-line development [13, 14]. Despite their presence and function being well-conserved, it is not unusual for piRNA sequences to vary across species [61], reflecting different exposures to TEs over time. In this case, it may also reflect an increased investment in host defense in the dam lines, which have a focus on reproductive traits. While the piRNA defense system is important for genomic integrity it can also sometimes be beneficial to allow transposon activation leading to genomic diversity through novel insertions. For instance, the insertion of a SINE element in the first intron of the *VRTN* gene has been linked to the presence of additional vertebrae in some pig breeds [62, 63]. Such variation may have contributed to the diversification of pig breeds over time and may also account for the patterns observed in our data.

In the sire lines, our results show higher occurrence of mt rsRNA than in dam lines. This difference could potentially influence traits such as growth and metabolism, as suggested by a previous study in which microinjections of nuclear rsRNA led to similar phenotypic changes [58]. The enriched levels of sRNAs originating from both mitochondrial rRNAs in sire lines could potentially be due to varying RNase levels. Previous studies have shown that knocking out epididymal-specific RNases, which cleave tRNAs and rRNAs, leads to subfertility in male mice and alters the sRNA profiles along with proteins involved in mitochondrial function and sperm energy generation [64]. Paternal environmental exposure such as dietary change, stress or toxicants changes the sRNA profiles in sperm during the epididymal passage [26, 30, 58]. During sperm maturation, nuclear sRNAs and miRNAs are loaded through epididymosomes to the sperm, while mitochondrial sRNAs and nuclear piRNAs originate within the spermatozoa [27]. In humans, we previously found several mitochondrial sRNA – including 12S and 16S rsRNA – upregulated following a high-sugar diet [65]. Similarly, in our *Drosophila* model of intergenerational metabolic response [35], a high-sugar diet increased sperm-borne levels of mitochondrial piRNA, rsRNA and tsRNA [66]. In mice, a high-fat diet induced elevated mt tsRNA levels in sperm and a metabolic phenotype in the offspring [27]. Further research is needed to evaluate if similar effects are evolutionary conserved in pigs.

Lastly, we performed a gene ontology enrichment analysis to discover the biological processes potentially associated with the upregulated miRNAs. The predicted processes involved were primarily linked to development and metabolic processes. The most prominently upregulated miRNA, mir-191, has previously been associated with high fertility rate and high-quality embryos in humans [67], and reportedly targets Sox4 [68], which is important for heart and neurodevelopment [69] and possibly influences white adipose tissue [70]. The let-7-family, which widely conserved across species [71], was also detected in our study and has been linked to spermatogenesis and embryogenesis [72].

The study presented has a number of limitations. First and foremost, given the exploratory nature of this study, our findings should be interpreted as descriptive and hypothesis-generating. While breed-specific clustering was observed, sperm-borne sRNA profiles are known to display high inter-individual variability, which together with our small sample size (six boars per breed), necessitates cautious interpretation and further validation.

Secondly, there are some methodological shortcomings as the sperm was not snap-frozen immediately after ejaculation, and samples spent time in transport and semen extender. However, this corresponds to the condition of the sperm sample when used for insemination by farmers. All samples appear to have similar integrity and yield, suggesting results should be comparable across breeds, and the results are similar to previous technical studies [53]. Another major challenge for interpreting the data derives from bioinformatical constraints as the limited annotation of sRNA sequences in pigs restricted our ability to fully describe their functional roles. By carrying out our own characterization of TEs across pig genomes and the use of different stringencies for matching, we demonstrate that, with regards to unannotated sequences, our results appear as robust and unbiased as current bioinformatic analysis allows for. There was considerable variation even between dam and sire breeds, so future work would benefit from an increased number of breeds and samples for each type.

Nevertheless, this is the first study of its kind to explore differences in sperm-borne sRNAs in pigs with diverse breeding objectives and serves as a basis for future experimental work to validate the findings and to examine the role of paternal diet in affecting offspring and further exploring the Paternal Origins of Health and Disease (POHaD) paradigm [73, 74].

## Conclusion

Understanding the sRNA payload in sperm before fertilization, along with their function in embryonic development, may help create strategies to promote healthier offspring. By exploring the baseline sRNA content in boar sperm, we establish a foundation for understanding their regulatory roles in reproduction and growth. Our findings highlight distinct sRNA profiles associated with different breeding objectives – intra-dam variation in piRNAs and their distinction from sire lines through mitochondrial sRNA – revealing potential epigenetic mechanisms underlying differences between sire and dam lines. This knowledge could be leveraged in selective breeding programs by moving beyond traditional genetic selection to incorporate inherited environmental influences on animal performance, thereby optimizing fertility and offspring health through targeted selection and delivery of beneficial sRNA signatures. However, establishing this mechanistically will require targeted functional studies.

## Supplementary Information

Below is the link to the electronic supplementary material.

**Figure S1 **General boar sperm profile comparison.(**a**) The overall breakdown of the sRNA composition in an overall boar sperm sample with no mismatches. (**b**) Comparison of levels (mean CPM) of sRNA between lines (Sire vs Dam) and breeds (Duroc, Hampshire, Landrace, Yorkshire) with 3 mismatches. * *p* < 0.05, ** *p* < 0.01, *** *p* <0.001. (PDF 45 KB)

**Figure S2** Dam line piRNA divergence in LTR- and SINE-origin.(**a**) Differential expression analysis between dam lines. (**b**) Number of sRNA from each biotype that are differentially expressed between Yorkshire and Landrace. (**c**) Repetitive element annotations in the genome of each breed made in RepeatMasker. (**d**) Comparison of levels (mean CPM) of origin of piRNAs from different repetitive elements (RE). ** *p* < 0.01. (PDF 5880 KB)

**Figure S3** Cover plots of mitochondrial tRNAs. Each line represents the breeds showing the difference in levels of mitochondrial tsRNAs. X-axis depicts the position on the genome. Ala= Alanine, Arg= Arginine, Asn= Asparagine, Asp= Aspartic acid, Cys=Cysteine, Glu= Glutamic acid, Gln= Glutamine, Gly= Glycine, His=Histidine, Ile= Isoleucine, Leu= Leucine-UUR (position: 3 845-3 919), Leu2= Leucine-CUN (position: 12 865-12 934), Lys= Lysine, Met=Methionine, Phe= Phenylalanine, Pro= Proline, Ser= Serine-UCN (position: 8 059-8 129), Ser2= Serine-AGY (position: 12 806-12 864), Thr=Threonine, Trp= Tryptophan, Tyr= Tyrosine, Val= Valine. (PDF 61 KB)

**Figure S4** Fold changes mir-191 and mir-28 Bar graphs showing fold change values of mir-191 (**a**) and mir-28 (**b**) from RT-qPCR. For each miRNA 4 technical replicas were used. Unpaired t-test was used for statistical testing.D = Duroc; Y = Yorkshire. * *p* < 0.05, ** *p* < 0.01. (PDF 30 KB)

Supplementary file5 (XLSX 156450 KB)

## Data Availability

The code used for this experiment is available at https://github.com/signeisacson/Asratian_Breed. Sequencing data have been deposited in the NCBI BioProject database with accession number: PRJNA1279106.

## Abbreviations

Adj p value: Adjusted p-value
Ago: Argonaute proteins
Ala: Alanine
Arg: Arginine
Asn: Asparagine
Asp: Aspartic acid
CASA: Computer Assisted Semen Analysis
CPM: Counts per million
Cys: Cysteine
D: Duroc
FC: Fold change
FDR: False discovery rate
Gln: Glutamine
Glu: Glutamic acid
Gly: Glycine
H: Hampshire
His: Histidine
i’-tRF: Internal tRNA-derived fragments
Ile: Isoleucine
L: Landrace
Leu: Leucine-UUR
Leu2: Leucine-CUN
LINE: Long interspersed nuclear elements
LTR: Long terminal repeat elements
Lys: Lysine
Met: Methionine
Min: Minutes
miRNA: microRNA
MitoPLD: Mammal phospholipase D
mt pc: Mitochondrial protein coding
mt piRNA: Mitochondrial PIWI-interacting RNA
mt rsRNA: Mitochondrial ribosomal RNA-derived small RNA
mt tsRNA: Mitochondrial transfer RNA-derived small RNA
mt: Mitochondrial
NA: Not Associated with repetitive elements
no anno: No annotation
NS: Not significant
PC: Principal components
pc: Protein coding
PCA: Principal component analysis
Phe: Phenylalanine
piRNA: PIWI-interacting RNA
POHaD: Paternal Origins of Health and Diseases
Pro: Proline
REs: Repetitive elements
RISC: RNA-induced silencing complex
rRNA: Ribosomal RNA
rsRNA: Ribosomal RNA-derived small RNA
Ser: Serine-UCN
Ser2: Serine-AGY
SINE: Short interspersed nuclear elements
snRNA: Small nuclear RNA
sRNA: Small non-coding RNA
srpRNA: Signal recognition particle RNA
TEs: Transposable elements
Thr: Threonine
tRFs: tRNA fragments
tRNA: Transfer RNA
Trp: Tryptophan
tsRNA: Transfer RNA-derived small RNA
Tyr: Tyrosine
Val: Valine
Y: Yorkshire
