# Supplementary figures and images for: Breed-specific divergence in boar sperm regulatory profiles involves piRNA and mitochondrial small RNAs
Source: Cell Mol Life Sci. 2026 Jun 4;83(1):243. doi: 10.1007/s00018-026-06244-8 (PMC13241569; doi:10.1007/s00018-026-06244-8)

**a** Overall sRNA composition, 0 mismatches

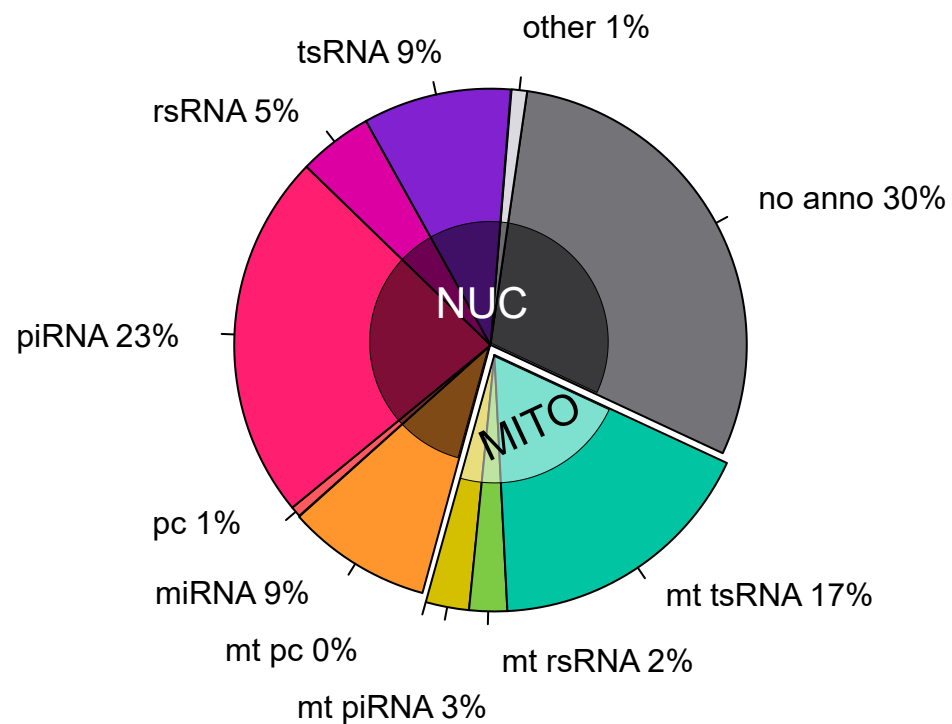

**b**

Line and breed comparisons

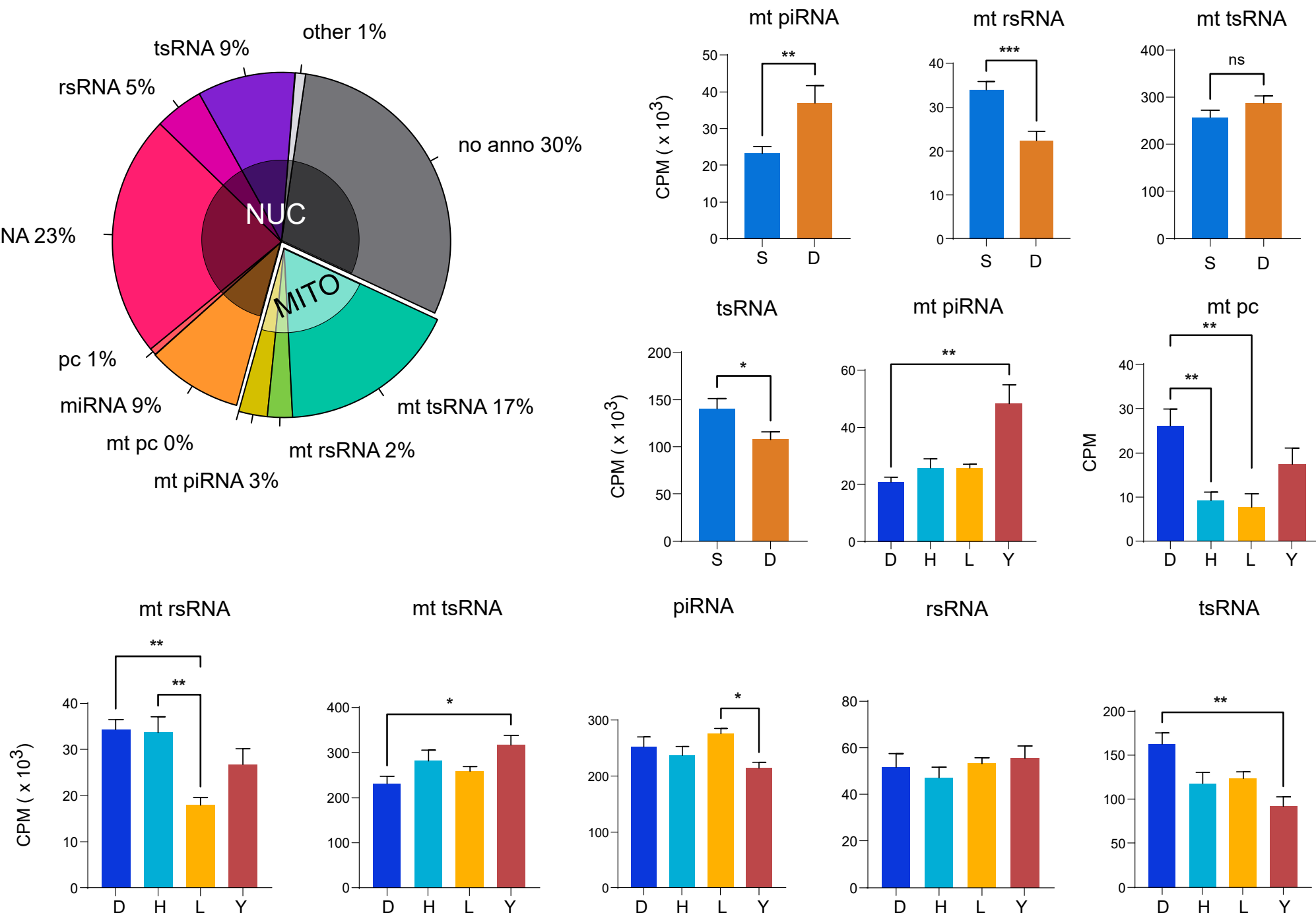

Supplement: Supplementary file 1 — Figure S1 General boar sperm profile comparison.(a) The overall breakdown of the sRNA composition in an overall boar sperm sample with no mismatches. (b) Comparison of levels (mean CPM) of sRNA between lines (Sire vs Dam) and breeds (Duroc, Hampshire, Landrace, Yorkshire) with 3 mismatches. * p < 0.05, ** p < 0.01, *** p <0.001. (PDF 45 KB) [file 18_2026_6244_MOESM1_ESM.pdf]

Duroc

Hampshire

Landrace

Yorkshire

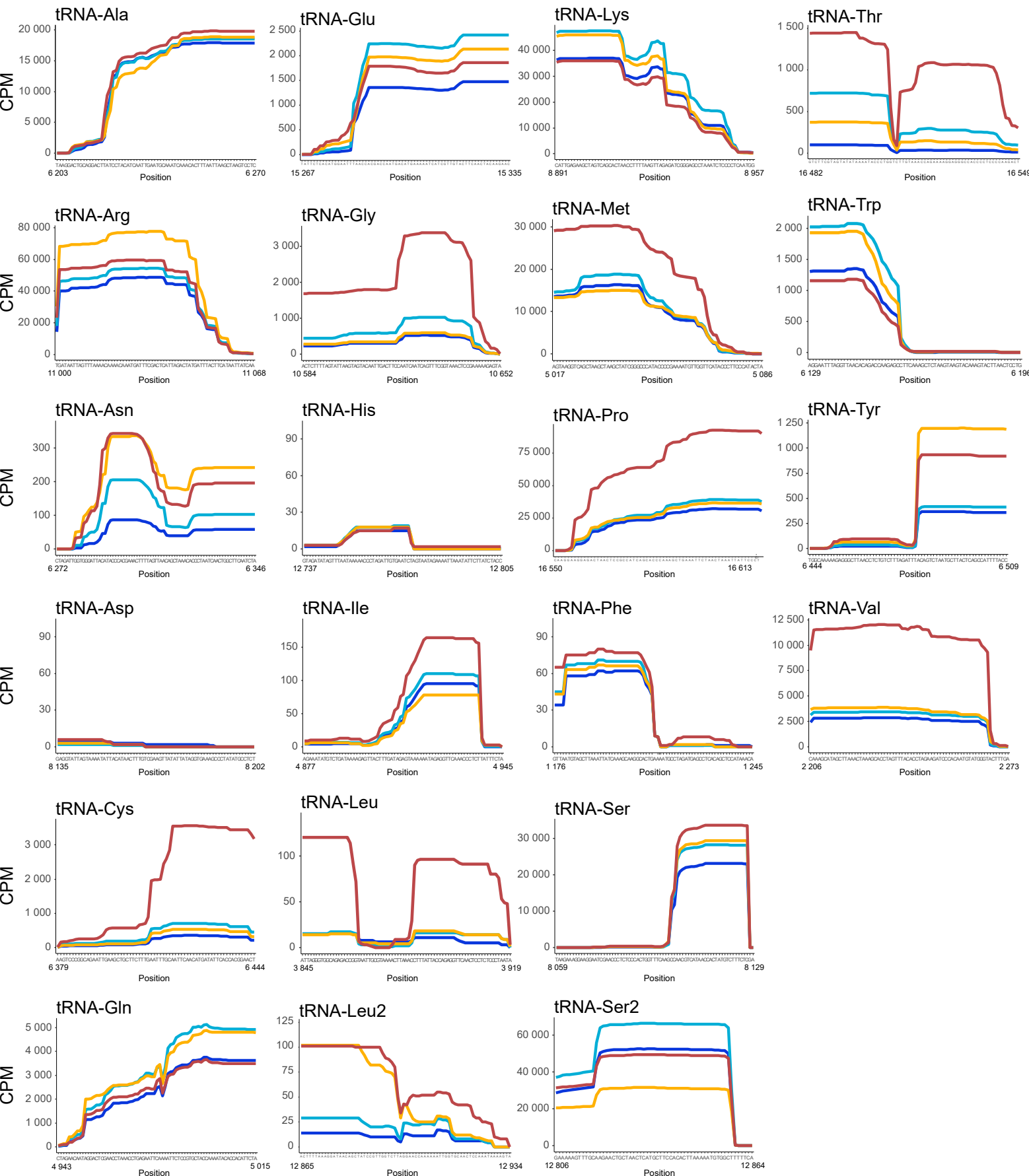

Supplement: Supplementary file 3 — Figure S3 Cover plots of mitochondrial tRNAs. Each line represents the breeds showing the difference in levels of mitochondrial tsRNAs. X-axis depicts the position on the genome. Ala= Alanine, Arg= Arginine, Asn= Asparagine, Asp= Aspartic acid, Cys=Cysteine, Glu= Glutamic acid, Gln= Glutamine, Gly= Glycine, His=Histidine, Ile= Isoleucine, Leu= Leucine-UUR (position: 3 845-3 919), Leu2= Leucine-CUN (position: 12 865-12 934), Lys= Lysine, Met=Methionine, Phe= Phenylalanine, Pro= Proline, Ser= Serine-UCN (position: 8 059-8 129), Ser2= Serine-AGY (position: 12 806-12 864), Thr=Threonine, Trp= Tryptophan, Tyr= Tyrosine, Val= Valine. (PDF 61 KB) [file 18_2026_6244_MOESM3_ESM.pdf]

a

mir-191

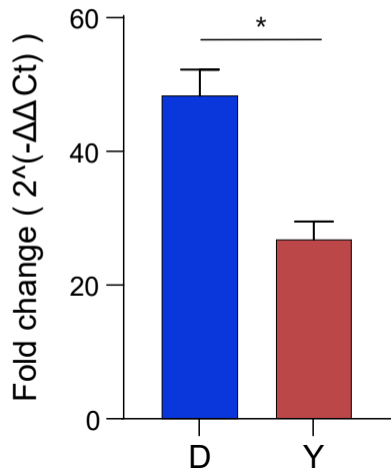

b

mir-28

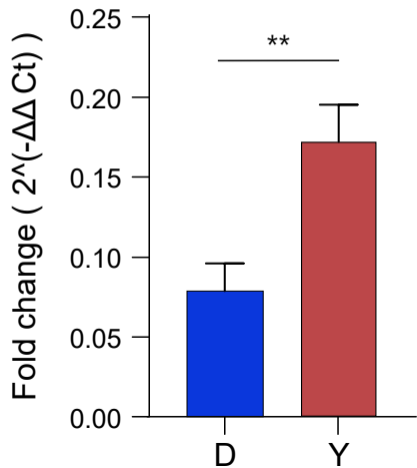

Supplement: Supplementary file 4 — Figure S4 Fold changes mir-191 and mir-28 Bar graphs showing fold change values of mir-191 (a) and mir-28 (b) from RT-qPCR. For each miRNA 4 technical replicas were used. Unpaired t-test was used for statistical testing.D = Duroc; Y = Yorkshire. * p < 0.05, ** p < 0.01. (PDF 30 KB) [file 18_2026_6244_MOESM4_ESM.pdf]
