# Supplementary material for: Breed-specific divergence in boar sperm regulatory profiles involves piRNA and mitochondrial small RNAs
Source: Cell Mol Life Sci. 2026 Jun 4;83(1):243. doi: 10.1007/s00018-026-06244-8 (PMC13241569; doi:10.1007/s00018-026-06244-8)
Supplement: Supplementary file 2 — Figure S2 Dam line piRNA divergence in LTR- and SINE-origin.(a) Differential expression analysis between dam lines. (b) Number of sRNA from each biotype that are differentially expressed between Yorkshire and Landrace. (c) Repetitive element annotations in the genome of each breed made in RepeatMasker. (d) Comparison of levels (mean CPM) of origin of piRNAs from different repetitive elements (RE). ** p < 0.01. (PDF 5880 KB) [file 18_2026_6244_MOESM2_ESM.pdf]

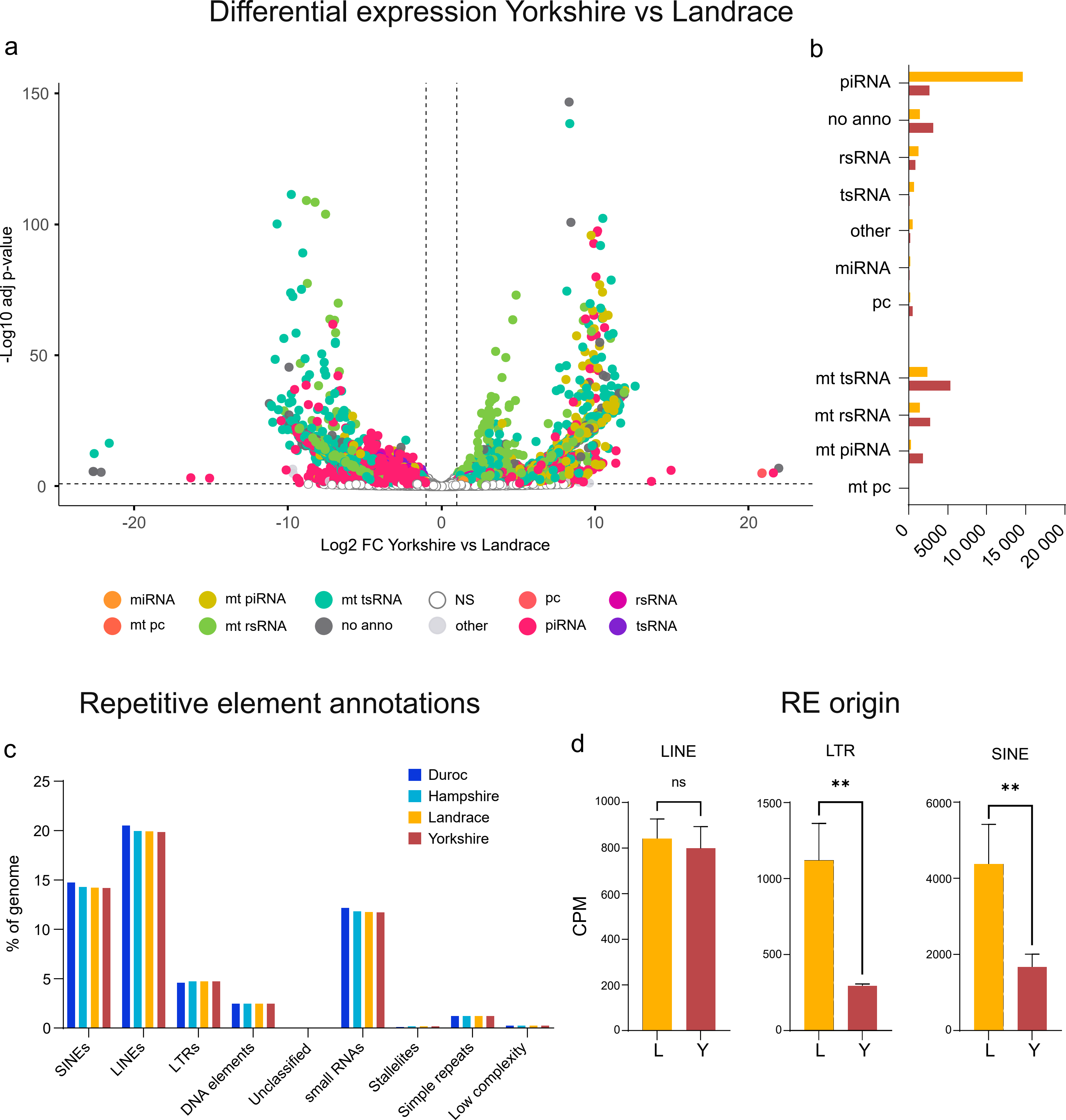

Differential expression Yorkshire vs Landrace
a
b
-Log10 adj p-value
Log2 FC Yorkshire vs Landrace
piRNA
no anno
rsRNA
tsRNA
other
miRNA
pc
mt tsRNA
mt rsRNA
mt piRNA
mt pc
miRNA
mt pc
mt piRNA
mt rsRNA
mt tsRNA
no anno
NS
other
pc
piRNA
rsRNA
tsRNA
Repetitive element annotations
RE origin
c
% of genome
Duroc
Hampshire
Landrace
Yorkshire
SINEs
LINEs
LTRs
DNA elements
Unclassified
small RNAs
Stallelites
Simple repeats
Low complexity
d
LINE
LTR
SINE
CPM
ns
**
**
L
Y
